# Sydenham Chorea Managed With Immunoglobulin in Acute Rheumatic Fever

**DOI:** 10.7759/cureus.14990

**Published:** 2021-05-12

**Authors:** Asim Ali, Gibson O Anugwom, Usama Rehman, Muhammad Zain Khalid, Mohammad Omar Saeeduddin

**Affiliations:** 1 Internal Medicine, Hayatabal Medical Complex, Peshawar, PAK; 2 Psychiatry and Behavioral Sciences, West Oaks Behavioral Hospital, Houston, USA; 3 Psychiatry and Behavioral Sciences, Houston Behavioral Healthcare Hospital, Houston, USA; 4 Department of Anaesthesia, Mayo Hospital, Lahore, PAK; 5 Internal Medicine, Liaquat National Hospital and Medical College, Karachi, PAK; 6 Psychiatry, Liaquat National Hospital and Medical College, Karachi, PAK

**Keywords:** sydenham chorea, acute rheumatic fever, intravenous immunoglobulin (ivig), choreiform movement, arf

## Abstract

Sydenham chorea (SC) is common in childhood with extensive differential diagnoses, including inherited disease, autoimmunity, endocrine disorders, and infections. SC due to acute rheumatic fever (ARF) is rare. Herein, we present a case of SC in an eight-year-old child who presented with choreiform movements of her face and limbs, including facial grimacing, difficulty walking, and slurred speech. She also had a runny nose and odynophagia. She had two episodes of sore throat in the last two months, and her physical examination was unremarkable except for hypertrophic tonsils and generalized hypotonia. Throat and blood culture were negative for group A streptococcus. Antistreptolysin O titer was 1139 IU/mL, and anti-deoxyribonuclease B titer was 2100 IU/mL, suggesting a recent group A streptococcal infection. Magnetic resonance imaging (MRI) of the brain revealed hyperintense signals in the thalami and corpus striatum. Echocardiogram was normal with no evidence of carditis. She was diagnosed with ARF and was commenced on amoxicillin and valproic acid. Later on, she was started on IVIG due to the persistence of chorea. Her symptoms improved, and she was discharged a week later on oral haloperidol for the next ten days.

## Introduction

Acute rheumatic fever (ARF) is a multisystem inflammatory disease that occurs as a delayed autoimmune response to group A beta-hemolytic streptococcal sore throat infection. ARF is generally seen in school-aged children and presents with migratory polyarthritis, Sydenham chorea (SC), carditis, erythema marginatum, and subcutaneous nodules occurring in various combinations [1]. SC is a self-limited devastating neurological presentation of ARF. SC is rare in young children, and migratory arthritis and carditis remain the common presentation in children [2]. SC is considered among the common causes of choreiform movements in children and requires more aggressive management. Herein, we present a case of ARF presented with SC as an initial presentation managed with intravenous immunoglobulin (IVIG).

## Case presentation

An eight-year-old girl was brought to the emergency department by her parents for sudden onset of involuntary movements of her limbs and face for the last four days. Involuntary movements involved shaking hands, difficulty walking, facial grimacing, and difficulty performing daily activities, including eating, speech, and bathing. These movements first started in lower limbs, followed by upper limb and face involvement on the same day, further worsened the following day. Associated symptoms included a runny nose, odynophagia, and mild arthralgia for the last week. She reported these jerky movements up to seven times per day. She had no history of trauma, loss of consciousness, neck stiffness, malar rash, or any family history of similar diseases, such as epilepsy and Huntington's disease. Her mother reported that she had two episodes of sore throat followed by pharyngitis and upper airway disease in the last two months, for which she received antibiotics and symptomatic treatment.

Initial evaluation showed an anxious-looking girl, well oriented to time, place, and person. Choreiform movements were evident in the limbs and face. Cranial nerves were intact except for slurred speech. The sensation was normal, and she had generalized hypotonia. She had hypertrophic tonsils with no signs of inflammation. The rest of her examination, including her cardiovascular system, was normal. Her temperature was 98 °F, blood pressure 110/75 mmHg, respiratory rate 20, heat rate 81 per minute, and oxygen saturation 98% at room air. Her initial investigations were unremarkable except for an elevated erythrocyte sedimentation rate (Table 1).

**Table 1:** Initial blood workup.

Parameter	Lab values	Reference range
White blood cells	9010/mm^3^	4000–11,000
	Neutrophils (60.01%)	45–75%
	Lymphocyte (33.9%)	18–45%
Hemoglobin	11.9 g/dL	14–17
Alanine aminotrasferase	32 IU/L	<36
Aspartate aminotransferase	37 IU/L	<35
Serum creatinine	0.7 mg/dL	0.7–1.2
Urea nitrogen	11 mg/dL	8–20
Thyroid-stimulating hormone	0.7 mIU/L	0.5–5.0
Erythrocyte sedimentation rate	24	<20
C-reactive protein	8 mg/dL	<0.2
Sodium	137 mmol/L	135–145
Potassium	3.9 mmol/L	3.5–5.0
Serum copper	11 µg/dL	10–15

**Figure 1:**
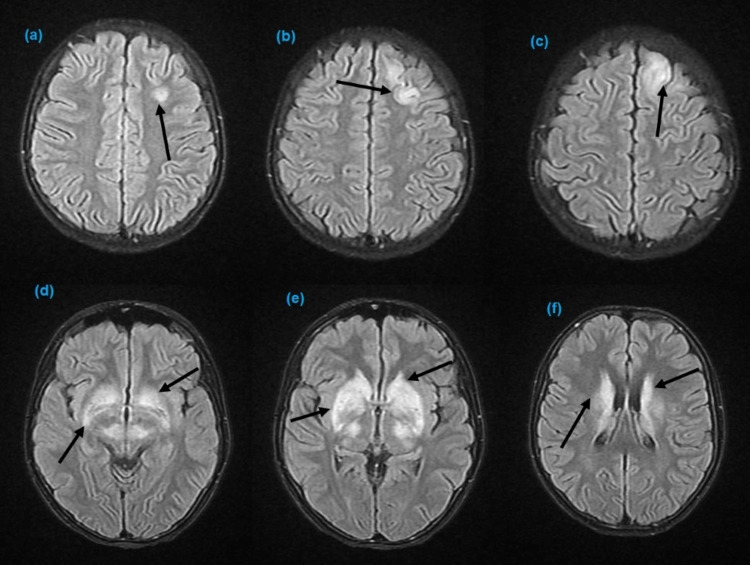
Axial FLAIR T-2 weighted images of the brain. (a–c) hyperintense signals in the left parietal lobe and (d–f) bilateral hyperintense signals in the thalami and corpus striatum. FLIAR: fluid-attenuated invention recovery.

Her blood culture was negative for group A streptococcus. Throat culture revealed commensal microbes. Detailed investigations, including antinuclear antibody, antiphospholipid antibodies, and antineutrophil cytoplasmic antibodies, were negative. Antistreptolysin O titer was 1139 IU/mL, and anti-deoxyribonuclease B titer was 2100 IU/mL, suggesting a recent group A streptococcal infection. Magnetic resonance imaging (MRI) of the brain was performed, which revealed bilateral symmetrical FLAIR (fluid-attenuated invention recovery) T-2 weighted hyperintense signals in thalami and corpus striatum, and altered signal intensity in the left parietal lobe (Figure 1). Her recent echocardiogram was normal with no evidence of carditis. She was diagnosed with SC due to ARF based on her clinical picture and ruling out other possible causes.

She was commenced on intravenous amoxicillin for five days. Her choreiform movements were managed with valproic acid 12.5 mg/kg/day. Improvements in condition were not observed. She was started on IVIG on day 5 due to the persistence of symptoms. She was discharged a week later on oral haloperidol of 0.25 mg/kg for the next 10 days and intramuscular injection penicillin G benzathine of 1.2 million units/2 ml every three weeks. Physiotherapy and speech therapy were also advised. Her condition improved significantly on her next visit after one month.

## Discussion

SC is one of the critical manifestations of ARF. SC is characterized by choreiform movements involving limbs and face and can also include muscle weakness, decreased muscle tone, and psychiatric symptoms. The severity of SC ranges from mild jerky movements to severe functional impairment, lasting from few days to months or years [3]. Although the incidence of SC had declined in developed countries, SC occurs approximately in one-third of the patients with ARF. A retrospective study from Nepal reported that 3.8% of ARF cases had chorea, and two-third of cases were females involving age from 10 to 16 years. Regmi et al. also reported that isolated SC was present in 0.6% of the patients with ARF, 0.8% with migratory arthritis, and 2.3% with carditis [4]. Our patient had isolated SC as a manifestation of ARF, making our case a unique entity. Another analysis from Israel reported SC in 24 patients. Most of the cases were reported in females, and 21% of patients presented with isolated chorea as an initial presentation of ARF [5].

Pathophysiology of multisystem involvement in ARF is believed to result from antibodies formed after streptococcal infection. SC occurs due to the molecular mimicry process that occurs in basal ganglia specifically [6]. Clinical evidence from the imaging modalities, including MRI, has revealed that basal ganglia are the primary cortical targets of streptococcal immune responses. Autoantibodies attack the basal ganglia responsible for choreiform movements. Antibodies formed against streptococcus bacterium cross-react with body antigens and target the group A carbohydrate (GAC) present on the bacterial surface, which is the immune dominant epitope of N-acetyl-beta-D-glucosamine (GlcNAc) [7]. These antibodies can target many host tissues and result in an autoimmune response. In SC, such antibodies cross-react with neuronal gangliosides (lysoganglioside GM1), an integral part of signal transduction in the nervous system and responsible for developmental and differential expression [7]. Many studies have suggested that autoantibodies enhance signal transduction in neural tissue by cross-reacting with lysoganglioside GM1, resulting in the release of excitatory neurotransmitters. Diagnosis of SC is clinical, and the patient generally has an acute onset of choreiform movements following a sore throat along with evidence of recent streptococcal infection and raised inflammatory markers. Neuroimaging is done to rule out other causes of chorea [8].

Management of SC involves supportive care, treatment of neuropsychiatric symptoms, elimination of group A streptococcus, and prophylaxis to prevent SC recurrence and rheumatic heart disease [1]. Medications used for the treatment of chorea include haloperidol (dopamine receptor antagonist) and antiepileptics (valproic acid or carbamazepine) [3]. However, the best treatment choice is not defined due to small size data, limited objective measures, and heterogeneous population. Another treatment option for chorea is IVIG, as neuronal antibodies are involved in the pathogenesis of SC. Clinical data also reported the use of IVIG for SC with favorable outcomes [9]. Prevention of SC is also essential, and secondary antibiotic prophylaxis is recommended to prevent neurologic and cardiac complications with streptococcus infection. Secondary prophylaxis for ARF is recommended up to 21 years of age by the Word Health Organization (WHO) [10].

Our patient had ARF, and SC was the first presentation. She was started on valproic acid; however, later, she was commenced on IVIG due to persistent chorea, resulting in the patient's recovery.

## Conclusions

SC is a rare presentation of ARF. Without adequate treatment and supportive care, SC can cause severe functional impairment. ARF should be considered as a differential diagnosis in school-aged children presenting with choreiform movements. Chorea can be managed with antiepileptics and antipsychotics; however, evidence on IVIG use for chorea has also been reported. There is no international consensus on the standard choice of treatment; therefore, IVIG should be used in severe functional impairment when unresponsive to other therapies.
